# The Hidden Link Between Endometriosis and Obesity: A State-of-the-Art Review

**DOI:** 10.7759/cureus.102896

**Published:** 2026-02-03

**Authors:** Shefali N Desai, Christina C Reed, Yamely Mendez, Qiannan Yang, Xiaoming Guan

**Affiliations:** 1 Obstetrics and Gynecology, Baylor College of Medicine, Houston, USA; 2 Research, Baylor College of Medicine, Houston, USA

**Keywords:** adipokines, adipose tissue, body mass index, endometriosis, glucagon-like peptide 1, inflammation, minimally invasive gynecological surgery, obesity

## Abstract

Endometriosis remains an under-researched disease with a wide range of symptoms. Endometriosis reduces a woman's quality of life and professional productivity, yet its exact causes, risk factors, and treatment have yet to be elucidated. Body mass index and endometriosis have been observed to be inversely related; however, this relationship may only be a correlation rather than a causation. Obesity may play a role in the inflammation and cell proliferation associated with endometriosis through the complex signaling pathways of adipokines. A literature review was done on endometriosis and obesity to gain insight and synthesize knowledge about opportunities to improve endometriosis care, its inflammatory pathogenesis, and the treatment potential of glucagon-like peptide-1. A Boolean search was performed via the Texas Medical Center Library with keywords including "endometriosis", "adipose tissue", "obesity", "adipokines", "glucagon-like peptide-1", and "inflammation". After screening titles, abstracts, and full texts, articles were excluded due to irrelevance, lack of access to full-text, and repetition. The literature revealed that at the onset of endometriosis symptoms in obese women, chronic pelvic pain and decreased appetite could cause misdiagnosis and weight loss, lowering the incidence of endometriosis in the obese population. Poor visualization, ventilatory compromise, and conversion to laparotomy during laparoscopy hinders diagnostic quality for obese patients. Interestingly, endometriosis and obesity share similar pathological markers, including leptin, adiponectin, tumor-necrosis-factor-α, and interleukin-6. Finally, glucagon-like peptide-1 was found to decrease pro-inflammatory secretions and macrophage infiltration.

## Introduction and background

The presence of endometrium-like tissue outside of the uterus defines endometriosis. It is characterized by a wide range of symptoms, including dysmenorrhea, pelvic pain, and abundant irregular menstruation. Endometriosis affects 10-15% of women of reproductive age [1].

Endometriosis is a chronic systemic disease. It affects metabolism in the liver and adipose tissue and can increase one's risk of cardiovascular disease [2]. Via alterations to gene expression, endometriosis can also increase pain sensitivity [3]. Lesions can be found in the peritoneal cavity, the ovaries, the bladder, and even distant places in the body, such as the lungs and the brain [1,4].

While the pathogenesis of endometriosis is still a mystery, several well-accepted theories exist. For example, Sampson's theory of retrograde menstruation, described for the first time in 1925 [5], says that menstrual blood can travel up the fallopian tubes and into the peritoneal cavity. This blood contains endometrial cells, which begin to proliferate around the abdominal organs. While this theory is well-known and logical, it does not explain the presence of deep infiltrating endometriosis or endometriosis outside the peritoneal cavity. Additionally, retrograde menstruation is a normal occurrence in women, yet it does not always lead to endometriosis [5]. An additional theory of the pathogenesis of endometriosis involves macrophages [5]. Macrophages play an important role in eliminating foreign cells and pathogens from the body. Interestingly, elevated macrophage counts have been found in ectopic endometrium. Macrophages can also secrete pro-inflammatory cytokines such as tumor necrosis factor alpha (TNF-α) and interleukin 6 (IL-6), which contribute to the proliferation of endometrial cells [5]. These cytokines can also be secreted by adipose tissue, leading to fat-induced inflammation in the peritoneal cavity [6].

Obesity is defined as having a body mass index (BMI) of 30 kg/m^2^ or higher. A long-term imbalance between calories consumed and calories expended is the main cause of obesity [7,8]. Complex combinations of genetic, socioeconomic, lifestyle, and cultural factors also contribute to this disease. Obesity can cause young individuals to have lower school attendance, resulting in decreased income potential. Along with the higher healthcare costs obesity can lead to, this puts economic strain on society [8]. An interesting characteristic of obesity is the inflammation associated with it. Adipose tissue secretes inflammatory signaling proteins, prompting investigation into the link between endometriosis and obesity. Like endometriosis, obesity is a complex, multifactorial disease [7].

It has been observed that endometriosis is associated with a low BMI [2]. However, this association is far from being understood and is surrounded by controversy [2]. Several hypotheses for this inverse correlation have been proposed that address obstacles in clinical and surgical care [9-12]. For example, the incidence of endometriosis in the obese population may be low due to diagnosis via laparoscopy having additional risks in obese patients [13]. However, it is unclear whether these are statistically significant. In fact, new technological innovations, such as minimally invasive gynecologic surgery, may be actively breaking barriers in gynecologic care [14]. The specific pathological mechanisms that may be involved in the connection between obesity and endometriosis have yet to be clarified [2]. Without a clear, definitive pathophysiologic explanation, it is difficult to settle controversy and confusion about the interplay between endometriosis and obesity. This review introduces information in a new light that suggests how obesity could increase the risk of endometriosis.

This intriguing connection could open doors to novel treatments for endometriosis. Inflammation has an established role in endometriosis, which presents opportunities for treatment with anti-inflammatory drugs [3]. For example, glucagon-like peptide-1 (GLP-1) is a multifaceted hormone with a wide range of treatment potential. It is currently used to treat obesity, type 2 diabetes, insulin resistance, and polycystic ovarian syndrome. GLP-1 also decreases inflammation and has anti-fibrotic properties, which could be promising for future treatment plans for endometriosis [15,16].

Considering the connections between endometriosis, obesity, inflammation, and GLP-1, this review of literature aims to highlight the clinical and inflammatory effects of excess adipose tissue and how GLP-1 may be able to decrease adipose tissue-induced inflammation in endometriosis.

## Review

Methods

**Figure 1:**
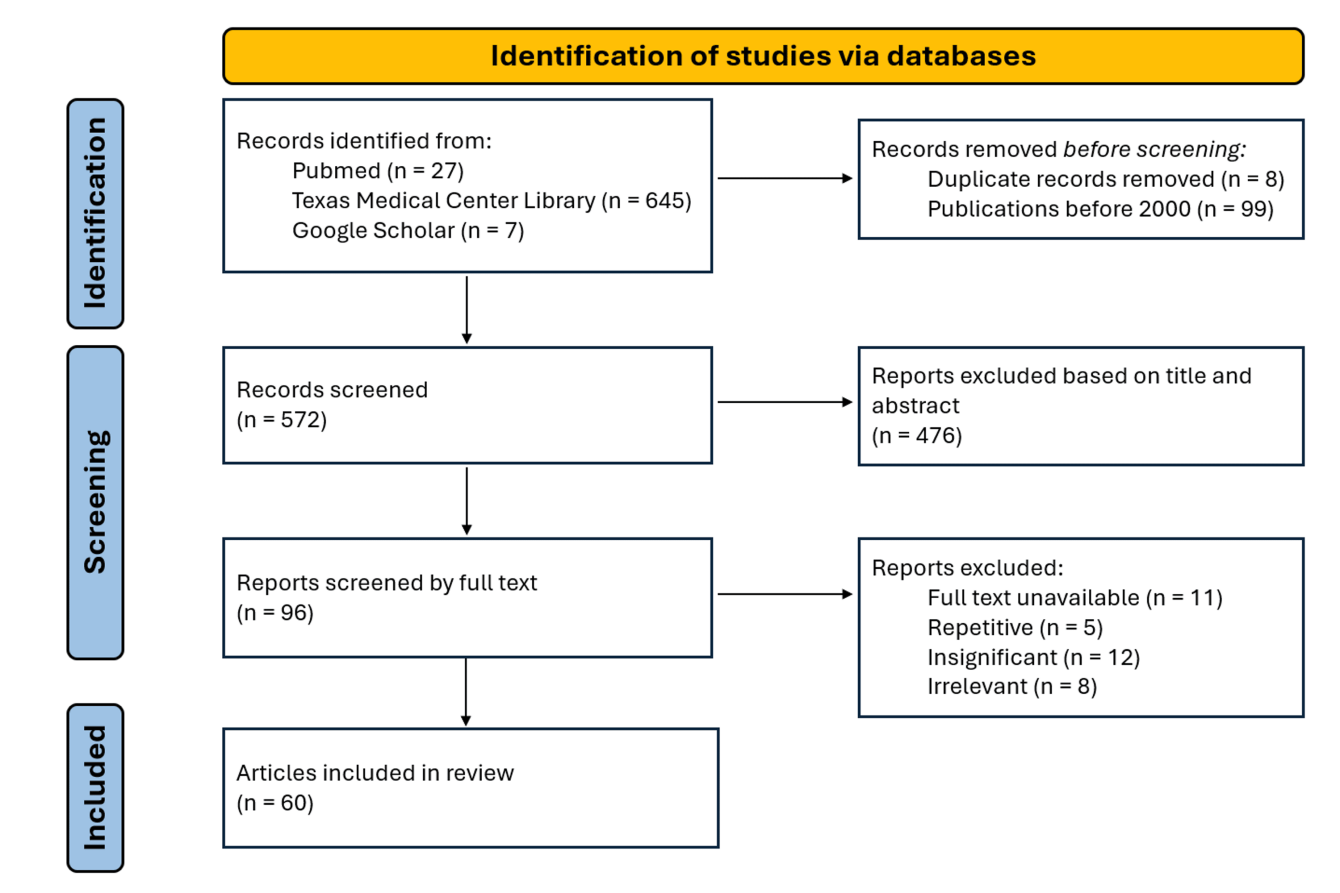
Preferred Reporting Items for Systematic Reviews and Meta-Analyses outlining the steps taken to collect the sources for this review.

A literature review was performed with articles gathered from the Texas Medical Center Library, PubMed Central, and Google Scholar. The Texas Medical Center Library includes articles from the following databases: AccessMedicine, BrowZine, CINAHL Plus, ClinicalKey, Cochrane Library, EMBASE, Journal Citation Reports, JoVE, Ovid, Psychiatry Online, PsycInfo, PubMed, RefWorks, VisualDx, and Web of Science. An advanced Boolean search was performed with different combinations of the following keywords: endometriosis, adipose tissue, clinical outcomes, GLP-1, adipokines, obesity, inflammation, minimally invasive gynecologic surgery, and BMI. Articles were eligible if they contained relevant information about obesity’s clinical, surgical, and/or pathological relationship with endometriosis, and/or GLP-1. Exclusion criteria included full-text unavailability, repetition, insignificance, and irrelevance. Bibliographical organization and duplicate removal were performed with Zotero, a reference management tool. Deduplication was blindly performed by one author. More details about the methodology can be found in Figure 1.

Obesity and endometriosis from a clinical perspective

Based on the review of literature performed, several reports of an inverse correlation between a woman's BMI and the incidence of endometriosis were found [9,17-22]. However, it is important to note that BMI is not an indicator of overall adipose tissue distribution, and it does not provide enough information to reflect the complexity of obesity [17,18]. In fact, women may weigh within a normal BMI range but be metabolically abnormal due to excess body fat or maldistributed adipose tissue. This is known as normal weight obesity syndrome, and it is a limitation on the reliability of BMI [20]. Additionally, there are several factors that could contribute to this correlation that are not endometriosis itself.

First, low BMI could be an effect of the disease rather than a risk factor or cause. Endometriosis is often diagnosed around seven years after the onset of symptoms [2]. This is important to recognize because chronic pelvic pain could reduce appetite and contribute to low BMIs at the time of diagnosis [18]. In seven years, a woman could lose a significant amount of weight and present to a physician at a much lower BMI than during the initial development of endometriosis. Interestingly, in a cohort of mice that were surgically integrated with endometriosis, their body weight was lower three weeks after surgery. The control group did not experience changes in body weight. Although the researchers did not elaborate on this finding, it proves to be interesting [23].

Secondly, patients with obesity face clinical obstacles in receiving care. Patients with obesity experience a delay in endometriosis diagnosis of an average of 18 months, while underweight or normal weight patients experience a delay of less than four months. These data were not influenced by race, ethnicity, or healthcare-seeking behavior. Patients of all weights showed a history of a similar incidence of therapy or emergency visits. Instead, this is because clinicians will often negate endometriosis as the cause of pelvic pain and other common symptoms of endometriosis. They will instead assume musculoskeletal or gastrointestinal disorders, as these are common conditions associated with obesity. As a result, patients with obesity have been historically undertreated for chronic pain [24].

Obesity and endometriosis from a surgical perspective

When patients with endometriosis have obesity, surgical obstacles and complexity are often present [9,11,24]. Healthcare providers are less likely to suggest laparoscopic intervention for patients with obesity. Performing a laparoscopy, searching for and excising endometriosis-like lesions, and doing a pathological analysis is considered the "golden standard diagnostic procedure" for endometriosis [2]. Therefore, the lower likelihood of this intervention being suggested leads to misdiagnosis, seemingly reducing the number of women with obesity who are diagnosed with endometriosis [12]. These women are often treated with medications rather than surgery, further leading to a lack of surgical diagnosis in the obese female population [9].

Before surgery can take place, preoperative imaging must occur to provide the surgical team with information on where endometriotic lesions are. Magnetic resonance imaging (MRI) and transvaginal ultrasounds (TVS) are valuable tools during this process. However, their value in mapping endometriotic lesions in patients with obesity remains an under-researched topic. It is known that TVS is a more accessible and cost-effective imaging technique [25]. TVS allows for the clear distinction between endometriotic implants and ovarian cysts [25] and has both a sensitivity and specificity of over 90% [26], but it is more invasive than MRI. Although the more costly option, MRIs can provide preoperative teams with more accurate information on disease localization and extension. With this imaging technique, visceral adipose tissue can be differentiated from endometriosis based on differences in their signaling intensities [25]. That being said, MRIs have a sensitivity of over 90%, but a specificity of only 79% [26]. This information includes both advantages and disadvantages of TVS and MRI as preoperative imaging techniques in the obese population. More research is needed to draw a conclusion on which method is best for women with endometriosis and obesity.

Also contributing to this lack of laparoscopic intervention in the obese female population is the compromise of visualization due to excess adipose tissue. Thankfully, there are several measures that can be taken to eliminate this obstacle and provide obese patients with the same standard of care as the general population. For example, the positions of the laparoscope ports can be changed and adapted to the patient. In obese patients, the location of the umbilicus is lower than the aortic bifurcation, so the umbilicus port can be placed higher to provide better visualization. Additional ports can also be added, such as a subpubic one, to increase visualization [27].

Additionally, there can be an increased risk of respiratory complications from laparoscopic surgery in patients with obesity. During laparoscopic surgery, the patient is in the Trendelenburg position, a downward angle of about 16 degrees. The patient's abdominal organs move towards the head, allowing for more visibility and access to the abdomen [28]. In patients with obesity, there can be ventilatory compromise in this position due to the excess mass pressing on organs in the chest. After sedation, the diaphragm and rib cage muscles relax. In the Trendelenburg position, this subjects the lungs to the entirety of the pressure from excess adipose tissue, potentially leading to ventilatory compromise. Discussion between the anesthesiologist and surgeon about an appropriate angle of depression can allow for good visibility without compromising respiration [29]. Aside from respiratory compromise, the Trendelenburg position can change surgical preparation in patients with obesity. The operating table must be able to support increased body mass and be wide enough to allow the patient's arms to be tucked to the side. This is a more ideal position for laparoscopic surgery because it decreases the risk of brachial plexus injury to the patient and lets the surgeon get closer to the patient. Finally, non-slip mattresses or adhesives can prevent the patient from sliding down the operating table [13].

Conversion to laparotomy, or open surgery, is a concerning possibility that increases in patients with obesity [13]. Open surgery increases blood loss, the need for transfusions, and post-operative complications [30]. In patients with BMIs over 35 kg/m^2^, there is a high conversion rate to laparotomy. This rate increases as BMI increases. Preoperatively, there may be a way to predict the occurrence of conversion to laparotomy. More visceral adipose tissue can cause a higher chance of conversion to laparotomy than fat around the legs can; therefore, assessment of adipose tissue distribution can be important to preoperative care and risk assessment [13].

There are also potential risks from difficult entry, wound infections, and bowel perforation [11,24,31]. Overall, women with obesity have historically had more postoperative and perioperative complications [11]. Out of the seven retrospective cohort analyses about this topic, six report findings that align with an increased risk for individuals with obesity [9-12,14,24,32]. The seventh study is an analysis specifically about endometriosis, and it only reports increased operative time for patients with obesity [32]. Surgeons are also becoming more trained in minimally invasive gynecologic surgery, making most perioperative and postoperative risks of laparoscopic surgery in patients with obesity insignificant [24].

With the development of technology and surgical techniques, robotic minimally invasive gynecologic surgery is increasing in popularity. The Da Vinci Surgical System is a robotic laparoscopic surgery machine that provides surgeons with 3-D high-definition viewing, increased instrument range of motion, and the ability to easily educate students. This method is best for patients with obesity because it combines the benefits of laparoscopic surgery with the benefits of an open laparotomy. Robotic surgery has lower blood loss, shorter postoperative recovery time, and an extremely low rate of postoperative complications [14].

While laparoscopic surgery may pose a series of risks to patients with obesity, this should not prevent patients from receiving excellent care. It is simply a matter of assessing the risks present and then adopting the necessary changes to ensure success and patient recovery. A summary of the risks and obstacles discussed can be found in Table 1.

**Table 1:** Reasons for an inverse correlation between endometriosis and obesity based on clinical, diagnostic, and surgical obstacles. BMI: body mass index References: [9,11,14,24,27,29]

Reason	Explanation
Chronic Pelvic Pain	Chronic pelvic pain leads to a decreased appetite and a low BMI.
Diagnostic Obstacles	Delay in diagnosis for patients with obesity compared to patients with normal weight. Pelvic pain is often thought to be the result of other conditions commonly observed in patients with obesity (musculoskeletal or gastrointestinal conditions).
Surgical Risks	Trendelenburg position can lead to ventilatory compromise. Additional risks include difficult entry with laparoscope, wound infections, and conversion to laparotomy.

Obesity and endometriosis from a pathological perspective

Obesity is a chronic inflammatory state characterized by excess adipose tissue. Adipose tissue, or fat, is the human body's primary source of stored energy. It is a key endocrine organ involved in a multitude of processes, including steroid production, immunoregulation, and reproduction. With increased adipose tissue comes increased adipokines. Adipokines are defined as any substance secreted by adipose tissue whether it is by infiltrating macrophages or adipocytes. These substances include cytokines, hormones, growth factors, and proteins associated with angiogenesis. Obesity is marked by a 40% increase in macrophage infiltration and an increase in proinflammatory cytokines. In fact, 30% of proteins expressed in white adipose tissue are related to inflammatory and macrophage-specific genes [6]. While endometriosis is overall an estrogen-dependent condition, estrogen is not sufficient to induce endometriosis [33].

**Figure 2:**
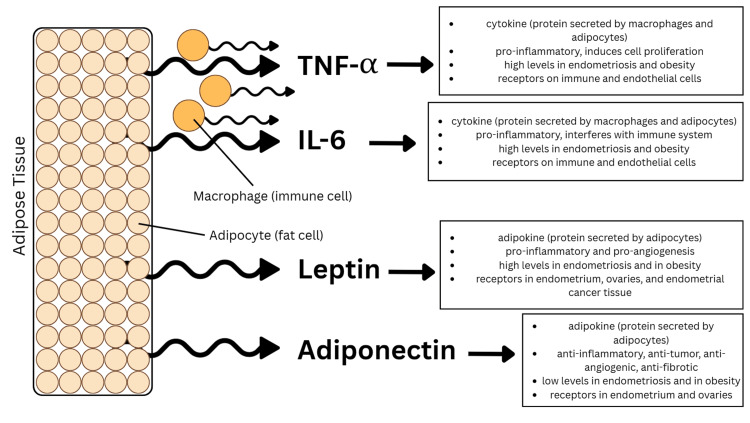
Overview of key adipokines in the pathogenesis of endometriosis. TNF-α: tumor necrosis factor alpha; IL-6: interleukin-6 References: [2,6,22,23,34]

There are two types of macrophages. M1 macrophages are pro-inflammatory, and M2 macrophages are anti-inflammatory [34]. Higher M1 macrophage counts are associated with obesity [17], and higher M2 macrophage counts are associated with leanness [18]. Macrophage counts are also elevated in the peritoneal fluid of patients with endometriosis [35]. In a 2021 pathological study of retroperitoneal adipose tissue, fat samples from around the uterus were collected from patients with and without endometriosis. Interestingly, more fibrosis and angiogenesis in the adipose tissue adjacent to the endometriotic lesions were found, and this tissue had more M1 macrophage infiltration. In other words, inflamed adipose tissue was adjacent to endometriotic lesions in all patients. In this same study, the overall macrophage expression levels were higher in endometriosis patients than in control patients [36]. This is important because macrophages also secrete pro-inflammatory cytokines such as TNF-α and IL-6 [6]. These cytokines, as well as additional molecules, will be discussed later in the article. An overview of these can be viewed in Figure 2.

Leptin

Leptin is an adipocyte-derived protein encoded by the obesity gene [37]. Since its discovery in 1994, leptin has been an essential part of understanding how adipose tissue communicates with other body systems [38]. Its primary functions are regulating food intake and energy consumption. After entering the brain via cerebrospinal fluid or, more frequently, a transport protein, leptin must target two main types of neurons in the hypothalamus. The first neuron type (POMC neurons), when targeted, secretes anorexigenic neuropeptides that suppress appetite. The second neuron type (AgRP/NPY neurons), when targeted, inhibits neuropeptides that normally stimulate appetite [39]. It can also promote cell growth and cell viability in certain areas [40]. Fat mass, gender, and insulin levels determine leptin levels in humans. In women, leptin levels are higher [41].

In normally functioning leptin signaling pathways, higher leptin levels should result in decreased energy intake and higher energy expenditure. Obesity is associated with higher leptin levels due to the presence of excess adipose tissue [42]. However, in individuals with obesity, high leptin levels are associated with leptin resistance [38]. Therefore, the ability of leptin to regulate appetite is suppressed in people with obesity [42]. Several mechanisms may underlie this phenomenon. For example, when leptin levels exceed 25-30 ng/mL, leptin concentrations in the brain stop increasing due to saturation of the transport system. This leads to increased levels of serum leptin, although they are not able to cause a physiological change. This accumulation of leptin can heighten its pro-inflammatory effects [39].

Out of the eight articles reviewed regarding leptin levels in endometriosis, eight reported significantly higher leptin levels in the peritoneal fluid of individuals with endometriosis [2,23,37,40,41,43-45]. For a given BMI, serum and peritoneal levels of leptin were also found to be significantly higher in subjects with endometriosis compared to controls. This suggests that, while BMI is still an important factor, there might be an effect of endometriosis on leptin levels independent of BMI [37]. Even the expression of the leptin receptor was increased in ectopic endometrial tissue compared to normal tissue, indicating that the heightened leptin signals are being received and causing a change in the body. Leptin receptors are found in the placenta, ovaries, endometrium, and endometrial cancer tissue. In fact, in a study conducted with ovarian endometriosis tissue samples in 2012, the knockdown of the leptin receptor gene impaired the ability of leptin to induce cell growth [40].

Leptin may play a key role in promoting inflammation and in the development of endometriosis. Leptin has pro-inflammatory and angiogenesis functions, both of which promote cell proliferation [23]. In fact, studies have shown that leptin can even be involved in secondary signaling pathways with other pro-inflammatory molecules. For example, leptin can promote the secretion of inflammatory and angiogenic factors or even be induced by substances such as TNF-α [40,41]. Interestingly, leptin concentrations have been found to be high in patients with stage I or II endometriosis and low in patients with advanced stage endometriosis. This could be an effect of differences in the pathogenesis of late and early-stage endometriosis. The finding of high concentrations of leptin in endometriosis has been repeatedly confirmed, so more studies are needed to assess the specific functions leptin has in inflammation [37,43]. The role of leptin in cellular maturation and growth begs the question of how it could be contributing to the development of endometriosis [41]. In a mouse model experiment performed in 2022, high-fat diet-induced and genetically modified mice were tested for leptin levels. These mice were integrated with fluorescently tagged endometrial lesions to assess the effect of leptin on endometriosis. The mice that were put on the high-fat diet revealed a significant increase in the development of endometriosis compared with controls. This conclusion was based on the fact that they had a higher number of ectopic lesions. These results support previous studies that report that obesity does not protect from endometriosis and may even be correlated with more severe stages of the disease [23].

Adiponectin

Adiponectin is an additional adipose tissue-secreted protein that influences insulin sensitivity and metabolic balance, as well as protects against type 2 diabetes [16,38,42]. Lower levels of adiponectin have been observed in obesity, and these low levels can lead to dyslipidemia. Adiponectin can also interfere with other signaling pathways, reducing the action of inflammatory cytokines such as TNF-α. Remarkably, adiponectin may also have a variety of anti-tumor effects, and it has been positively correlated with a decreased risk of breast cancer and endometrial cancer [38]. As far as adiponectin's correlation with endometriosis, lower levels of adiponectin have been found in patients with endometriosis [18-20,43]. This coincides with the effect of obesity on endometriosis, indicating a possible relationship between obesity, adiponectin, and endometriosis. There are adiponectin receptors in the endometrium, ovaries, and even the placenta, which makes the presence of adiponectin signaling pathways in endometriosis a feasible hypothesis [6]. A study in 2021 analyzing the circulating and peritoneal levels of leptin and adiponectin hypothesized that adiponectin may play a protective role in endometriosis by inhibiting pathogenic factors in the disease and decreasing the chance of angiogenesis and inflammation [43]. Adiponectin is truly a fascinating and promising substance based on its anti-inflammatory, anti-angiogenic, anti-fibrotic, and anti-tumor properties [38,43,46].

Tumor Necrosis Factor Alpha

TNF-α is a cytokine produced by macrophages, adipocytes, T cells, and endothelial cells. TNF-α receptors can be found in immune and endothelial cells. This protein participates in vasodilation and edema formation, induces fevers, and contributes to oxidative stress in inflammation sites. Vasodilation leads to flare and high temperatures at an inflamed site, leading to systemic inflammation. Edema is the consequence of high vessel permeability and increased hydrostatic pressure in capillaries [47]. High levels of TNF-α have been found in individuals with obesity and endometriosis. These levels increase with the severity of endometriosis [46,48]. In endometriosis, macrophages are recruited to the peritoneal cavity, contributing to the higher concentration of TNF-α [48]. Additionally, TNF-α can disrupt the structure of the endothelium of the vessel. TNF-α degrades the protective layer of the endothelium called the glycocalyx layer. This layer helps to regulate inflammation [47].

TNF-α is pro-inflammatory and has a role in the immune system during inflammation. It also increases cell proliferation and differentiation [44]. This allows it to contribute to the adhesion of endometrial cells to other tissues and to the angiogenesis seen in endometriosis [48]. Interestingly, TNF-α increases the synthesis of anti-inflammatory factors through a negative feedback loop [47]. This raises the question of how endometriosis might be disrupting this pathway or if the initial disruption can lead to endometriosis.

Matrix metalloproteinases (MMPs) are a group of enzymes expressed during menstrual breakdown and during the estrogen-mediated growth of endometrial tissue. TNF-α can upregulate several MMPs, which could contribute to the increased invasiveness of uterine endometrial fragments in endometriosis. Additionally, inhibitors of MMPs are decreased in endometriosis [48].

In a baboon model reviewed in 2019, the blockage of TNF-α using a recombinant TNF receptor or an antibody against TNF prevented the establishment and the size of endometriosis lesions. This was because the recombinant receptor bonded to the free-floating TNF-α and prevented it from moving and activating pathways [4].

Interleukin-6

IL-6 is a multifunctional cytokine predominantly secreted by macrophages, although adipose tissue does contribute to 35% of circulating IL-6 [49]. With increased macrophage counts in women with endometriosis (p<0.05), IL-6 is increased in the peritoneal fluid of women with endometriosis, and they increase with disease severity [49,50]. IL-6 levels are also increased in individuals with obesity [38]. There are two forms of the IL-6 receptor: membrane-bound and soluble. There is a higher cell membrane and soluble receptor count in women with endometriosis, but the soluble receptor count does not change between different severities of endometriosis. IL-6 is thought to promote endometriosis through pro-inflammatory and immune function interference. Specifically, IL-6 forms a positive feedback loop with haptoglobin. This means that as IL-6 levels increase, haptoglobin levels increase. Haptoglobin is a protein that helps ectopic endometrial cells escape from immune cells and avoid elimination by adhering to the surface of macrophages [50].

Glucagon-like peptide-1 and its anti-inflammatory properties

GLP-1 was first discovered in 1987 [51]. It is a polypeptide chain of 30 amino acids and is categorized as an incretin hormone because it is secreted from L cells in response to nutrient ingestion [52,53]. GLP-1 primarily activates pathways important in reducing appetite and glucose metabolism [16,51]. It helps regulate the secretion of insulin and can improve insulin sensitivity by enhancing glucose-dependent insulin production and secretion, decreasing glucagon secretion, and increasing glucose uptake and glycogen synthesis [51,54]. Therefore, the importance of GLP-1 in type two diabetes and weight loss has been established [51]. The question lies in how the secondary effects of GLP-1 may pave the way for a new endometriosis therapy.

In recent years, GLP-1 has been proven to possess properties that may be helpful in mitigating endometriosis. For example, GLP-1 reduces the production and secretion of pro-inflammatory cytokines such as TNF-α and IL-6. In a review written in 2016, a study showed that GLP-1 therapy reduced the production of TNF-α and IL-6, two cytokines already proven to have inflammatory properties [52]. There is even evidence to prove that GLP-1 downregulates the expression of IL-6 [55]. Additionally, in a mouse model reviewed in 2022, GLP-1 was observed to reduce systemic plasma levels of TNF-α and IL-6 [56].

Another example of the anti-inflammatory effects of GLP-1 is demonstrated in its ability to decrease macrophage-induced inflammation. In patients with obesity, GLP-1 levels are lower. Lower levels of GLP-1 lead to fewer CD86 markers on macrophages. These markers are essential in the elimination of endometrial cells after retrograde menstruation. For this reason, decreased GLP-1 levels may contribute to the development of endometriosis. In fact, GLP-1 levels in the peritoneal fluid of patients with endometriosis are significantly lower (p=0.009) [57]. High levels of GLP-1 may even be associated with a lower risk of developing endometrial cancer and other hormone-sensitive cancers [16]. Interestingly, GLP-1-like molecules have even been found to reduce macrophage infiltration, decrease M1 (inflammatory) markers, and increase M2 (anti-inflammatory) markers. This leads to decreased pro-inflammatory secretions and increased anti-inflammatory secretions [55,56]. GLP-1 can also help counteract the inhibition of adiponectin that macrophage infiltration causes [55].

**Figure 3:**
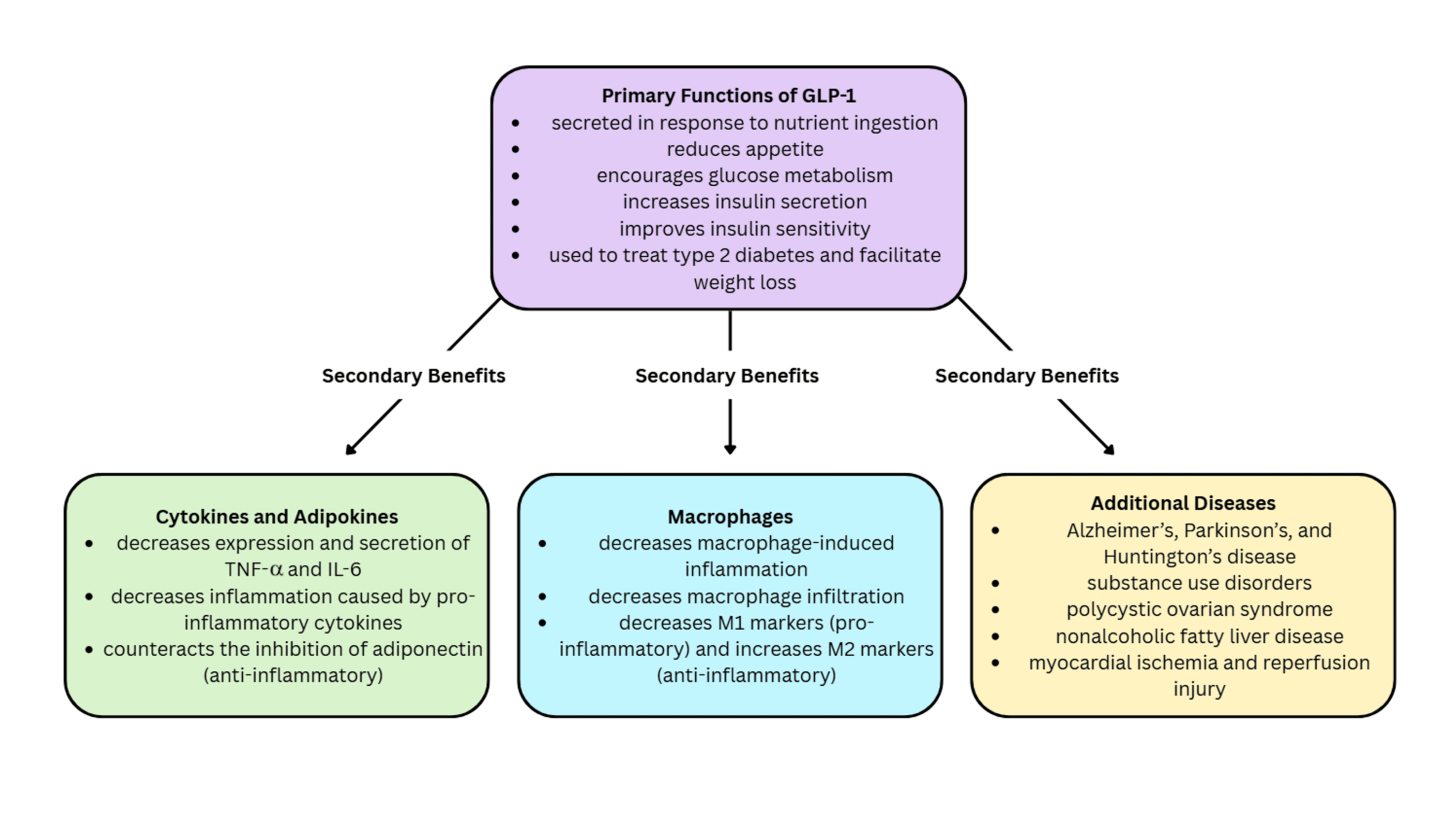
GLP-1 and its primary and secondary benefits. GLP-1: glucagon-like peptide-1; TNF-α: tumor necrosis factor alpha; IL-6: interleukin-6 References: [16,51-53,56,57]

Up to 30-50% of women with endometriosis may experience infertility [1]. GLP-1 receptors exist in endometrial tissue and epithelial cells [56]. It can help improve female fertility and endometrial receptivity by mitigating the effects of endometriosis via anti-inflammatory or anti-fibrotic pathways [16,54]. For example, liraglutide and exenatide are conformational modifications of GLP-1 that have been used to lower inflammation in polycystic ovarian syndrome and restore normal functions of the female reproductive system [58,59]. A summary of GLP-1 and its benefits can be seen in Figure 3.

Discussion

The role of clinical obstacles, adipokine/cytokine signaling, and the emerging potential of GLP-1 opens doors for new breakthroughs in endometriosis research. In contradiction to observed relationships between obesity and endometriosis, there is significant pathological evidence that suggests obesity and endometriosis may be positively correlated. With further research, obesity may become a risk factor for endometriosis. To improve current knowledge, the significance of clinical and surgical obstacles for women with obesity on the inverse correlation between BMI and the incidence of endometriosis needs further elucidation. These findings have the potential to improve care for women with endometriosis and obesity. Studies that analyze metrics beyond BMI, such as adipose tissue distribution and metabolic function, can reveal overlooked factors in endometriosis. Several studies reviewed here have presented conflicting results regarding BMI, so this could reveal any confounding variables contributing to this variation. The role of adipokines in the development of endometriosis has proven to be significant, and their connections to GLP-1 may lead to new treatment options for endometriosis. Leptin resistance is a promising factor for further research due to its ability to produce contradictory levels of leptin and metabolic responses.

Out of the 60 articles reviewed, eight articles reported small sample sizes or limited data. This was the most common limitation among the original research articles reviewed. Additional limitations included outdated equipment, patient-reported data, and patient-selection biases. These factors could be contributing to the variation and contradiction observed in several studies. Designing studies with larger sample sizes and less bias could eliminate any confounding variables.

Research gaps and future directions

Research gaps in the current literature include a lack of understanding of the molecular and biological mechanisms involved in endometriosis. This would be fundamental to treating endometriosis and preventing it at the beginning of its development [2,12]. Additionally, more in vivo and in vitro models are needed to study the characteristics of the active phase of endometriosis in humans and the role of leptin in pelvic pain [40]. Research is also needed on the molecular and cellular mechanisms and signals in adipose tissue [46]. Furthermore, how GLP-1 treatments can induce molecular changes in endometrial tissue and in adipose tissue has yet to be understood [16,55]. Clinical trials evaluating the effects of GLP-1 could test our hypothesis. Full elucidation about the specific effects of a large variety of adipokines, such as apelin, resistin, and chemerin, would be beneficial [42,60].

Future directions include investigating the different types and benefits of non-steroidal anti-inflammatory drugs as treatment options for endometriosis [4]. Additionally, further studies should include more measurements beyond BMI and consider adipose tissue distribution. These studies could utilize MRI or DEXA scans to assess differences between subcutaneous fat and visceral fat [17]. More emphasis on BMI stratification and the large range of body types could lead to new discoveries about higher BMI classes and their risk of developing endometriosis. Most of the studies reviewed here did not include women with BMIs in class II or III. Even more research on patients with low BMIs and how their immune systems may be altered is also needed [57]. Finally, as endometriosis can be found in several different places in the body, research focusing on the different lesion locations and how they may cause completely different pathological results could be beneficial [49].

## Conclusions

This literature review paves the road to a new understanding of endometriosis in women with obesity. Clinical obstacles and surgical challenges may be at play, affecting the incidence of endometriosis in the obese population. It is important to be aware of this in order to improve care for these patients. The inflammation associated with the development of endometriosis can be further understood in the context of adipokines and cytokines. Leptin, adiponectin, TNF-α, and IL-6 can influence the pathogenesis of endometriosis through their complex signaling pathways. Finally, GLP-1, its analogues, and its receptor agonists have high potential to improve endometriosis due to their wide range of secondary benefits.

More research is needed to conduct human clinical trials, understand the biological mechanisms of endometriosis and adipose tissue, and investigate the molecular changes that GLP-1 can induce. Nonetheless, these results highlight the exciting future possibilities for endometriosis research and treatment.
